# Fishing for the Virome of Tropical Tuna

**DOI:** 10.3390/v13071291

**Published:** 2021-07-02

**Authors:** Elsa Gadoin, Christelle Desnues, Sonia Monteil-Bouchard, Thierry Bouvier, Jean-Christophe Auguet, Emmanuelle Roque d’Orbcastel, Yvan Bettarel

**Affiliations:** 1 MARBEC, Université de Montpellier, CNRS, Ifremer, IRD, 34095 Montpellier, France; thierry.bouvier@cnrs.fr (T.B.); Jean-christophe.AUGUET@cnrs.fr (J.-C.A.); emmanuelle.roque@ifremer.fr (E.R.d.); yvan.bettarel@ird.fr (Y.B.); 2 Mediterranean Institute of Oceanography (MIO), 13009 Marseille, France; christelle.desnues@univ-amu.fr (C.D.); sonia.monteil@univ-amu.fr (S.M.-B.)

**Keywords:** tuna, virome diversity, microbiome

## Abstract

While planktonic viruses have received much attention in recent decades, knowledge of the virome of marine organisms, especially fish, still remains rudimentary. This is notably the case with tuna, which are among the most consumed fish worldwide and represent considerable economic, social and nutritional value. Yet the composition of the tuna virome and its biological and environmental determinants remain unknown. To begin to address this gap, we investigated the taxonomic diversity of viral communities inhabiting the skin mucus, gut and liver of two major tropical tuna species (skipjack and yellowfin) in individuals fished in the Atlantic and Indian Oceans. While we found significant differences in the virome composition between the organs, this was totally independent of the tuna species or sex. The tuna virome was mainly dominated by eukaryotic viruses in the digestive organs (gut and liver), while bacteriophages were predominant in the mucus. We observed the presence of specific viral families in each organ, some previously identified as fish or human pathogens (e.g., *Iridoviridae, Parvoviridae*, *Alloherpesviridae*, *Papillomaviridae*). Interestingly, we also detected a ‘core virome’ that was shared by all the organs and was mainly composed of *Caudovirales*, *Microviridae* and *Circoviridae*. These results show that tuna host a mosaic of viral niches, whose establishment, role and circulation remain to be elucidated.

## 1. Introduction

Marine animals live in close and complex interactions with an abundant and diverse assemblage of viruses, typically made up of bacteriophages and eukaryotic viruses. Both are suspected to play an essential role in the health of the host, either by contributing to its immune system [1,2] or, conversely, by transmitting infections in tissues [3]. In recent years, only a handful of studies have explored the virome of marine invertebrates such as corals [4,5,6] or sponges [7], while those studying marine vertebrates are even scarcer [8,9]. This is particularly true for marine fish, the viromes of which the ecological determinants, variability between species, organs, sex, areas of distribution, as well as risks and benefits to the host, are virtually unknown.

Tuna are an interesting model for investigating the fish virome since they are (i) a keystone species and top predators in pelagic ecosystems, (ii) one of the most consumed fish species worldwide [10], and (iii) microbiologically sensitive, as the consumption of tuna presents a risk of food poisoning linked to the presence of pathogenic—histamine-producing—bacteria [11].

In this study, we aimed to describe the taxonomic diversity of viral communities inhabiting the skin mucus, gut and liver of two major tropical tuna species (skipjack and yellowfin) in individuals of similar sizes sampled in the Atlantic and Indian Oceans. We sought to identify shared and specific viral taxa in these three key organs and to characterize the influences of species, sex and sampling site on the composition of their virome.

## 2. Materials and Methods

### 2.1. Sampling Procedure

*Tuna.* All skipjack (*Katsuwonus pelamis,* SKJ) and yellowfin (*Thunnus albacares,* YLF) tuna used in this study were sampled around fish aggregating devices (FAD), in the Atlantic (Ivory Coast, Gulf of Guinea, N04°55′00″, W03°42′19.97) and Indian Oceans (Reunion Island, S20°57′816″, E55°04′457″), during campaigns in July (10–11) and September (26–29) 2018, respectively. In total, 21 tuna were sampled in the Gulf of Guinea (6 skipjack and 15 yellowfin) and 27 off Reunion Island (18 skipjack and 9 yellowfin). In both locations the sampling and euthanasia of the fish were performed by professional fishermen working for the French National Research Institute for Sustainable Development (IRD) Exploited Tropical Pelagic Ecosystems Observatory (certified ISO 9001:2015). A hook and line were used, and the tuna always handled by the mouth using a clamp; euthanasia was performed by cervical dislocation (following European directive 2010/63/UE) immediately after capture. All participants wore gloves to avoid contamination during sampling.

### 2.2. Sampling of the Skin Mucus, Gut and Liver

*Skin mucus layer*. The superficial mucus layer was sampled immediately after the death by swabbing one side of the body surface, which remained untouched. Avoiding the head, which was manipulated during euthanasia, buccal swabs (Isohelix^®^, Harrietsham, UK SK-2S swabs) were rubbed from the back of the operculum to the caudal peduncle.

*Gastrointestinal content*. After the sampling of the skin mucus, fish were individually placed in plastic bags and immediately stored on ice before dissection (within 5 h after sampling). Back in the laboratory, each individual was dissected using sterile tools: the gastrointestinal tract was extracted and cut from below the stomach to the rectum. Placed on a sterile surface, the content of each gut was expelled by squeezing, and the collected contents (minimum volume of 5 mL) were homogenized before sampling.

*Liver*. Using a sterile cutter and forceps, a longitudinal piece of about 1 × 0.2 × 0.2 cm was cut from the right lobe (the largest) of the liver in each individual tuna. To avoid contamination from any other internal organs or fluids, the collected liver samples were rinsed with distilled water (through 0.2-µm filters).

*Storage.* All skin mucus, gut and liver samples were placed in 5 mL sterile cryovials, frozen in liquid nitrogen and stored at −80 °C in the laboratory until nucleic acid extraction.

### 2.3. Viral Nucleic Acid Extraction and Sequencing

*Samples preparation*. Three individuals (triplicate) of each sex (male and female) of each species (skipjack and tuna) in each ocean (Atlantic and Indian) were selected (a total of *n* = 3 indiv × 2 sex × 2 species × 2 oceans = 24 indiv). In each of the 24 tuna, skin mucus, gut and liver viral communities were sampled as described above (for a total of *n* = 3 organs × 24 indiv = 72 biological samples).

To separate virus from bacteria and organic matter, all biological samples (*n* = 72) were suspended in 0.02 μm filtered PBS. For gut (*n* = 24) and liver (*n* = 24), 1 g sub-samples were placed in 2 mL Eppendorf and completed with 0.02 μm filtered PBS. For skin mucus samples (*n* = 24), individual swabs were placed in 1.5 mL Eppendorf with 500 μL of 0.02 μm filtered PBS. Gut and skin mucus samples were vortexed at maximum speed, respectively, 10 and 5 min, while liver samples were crushed with a FatsPrep for 45 s at 6 ms/ms. All samples were then centrifugated 10 min at 1500× *g* at 4 °C and supernatants were collected. After a second centrifugation at 14,000× *g* at 4 °C for 15 min, supernatants were collected and put in clean Eppendorf (protocol adapted from Monteil-Bouchard et al., 2018) [12]. All resuspended samples were then digested 1 h at 37 °C with a cocktail of enzymes composed of 100 μL of 10X TurboDNase buffer (^®^Invitrogen, Carlsbad, CA, USA), 20 μL of TurboDNase (2 U/μL, ^®^Invitrogen), 5 μL of RNase A (3.6 U/μL, ^®^Macherey Nagel, Düren, Germany), 5 μL of Exonuclease I (20 U/μL, ^®^ThermoFisher Scientific, Waltham, MA, USA) and 5 μL of Benzonase (25 U/μL, ^®^Merck Millipore, Molsheim, France) for 1 mL of sample [12].

*Nucleic acid extraction*. Viral nucleic acids were extracted from each sample (*n* = 72) using the Roche High Pure Viral Nucleic acid extraction kit (^®^Roche Diagnostics, Meylan, France) following the manufacturer’s protocols. Following the extraction, 1 μL of RNase OUT (40 U/μL, ^®^Life Technologies, Saint Aubin, France) was added in all samples, then nucleic acid concentration was assessed using Qubit dsDNA high sensitivity (^®^Life Technologies), following the manufacturer’s instructions.

After quantification, triplicate individuals were pooled prior to the sequencing. Subsamples of 100 ng of extracted nucleic acid were taken from each of the three individuals replicates and pooled. Thus, for each organ a single replicate per sex (male and female), per species (skipjack and yellowfin) and per sampling site (Atlantic or Indian ocean) was obtained, for a total of 24 samples to be sequenced (n = 3 organs × 2 species × 2 sex × 2 sampling site = 24 samples).

*DNA amplification and purification*. DNA amplification was then performed in duplicate, using Genomiphi V3 kit (^®^GE Healthcare, Vélizy-Villacoublay, France) following the manufacturer’s protocols. Duplicate amplifications of each sample were pooled, diluted with 60 μL of biomolecular water and purified using NucleoFast plates (^®^Macherey Nagel). Plates were centrifugated 30 min at 4000× *g*, 100 μL of molecular water were added in each well before a second 30 min centrifugation at 4000× *g*. After the addition of 30 μL of molecular water in each well, plates were agitated during 10 min before the collection of the eluded sampled placed in clean Eppendorf. Samples were sequenced using the Illumina MiSeq Technology with Nextera XT library kit in 2 × 250 bp format.

### 2.4. Viral Sequences Treatment and Analysis

A total of 70,552,236 raw paired reads were obtained and trimmed, filtered, mapped and assembled into contigs/scaffolds using CLCgenomics7. Trimming parameters allowed the selection of sequences with a minimum length of 50 nucleotides and a quality above 0.05, resulting in 70,124,886 trimmed paired-reads. After assembly, the 828,771 contigs, with an average length of 596 bp (range 17 bp–117,982 bp), were aligned (BLASTx) against the *nr* database using DIAMOND [13]. The taxonomical annotations were then determined with MEGAN (LCA top percent = 0.1) [14]. Contigs assigned as bacteria or eukaryotes as well as non-assigned contigs were discarded and only the contigs assigned as viruses were analyzed (107,657 contigs). Viral sequences assigned as unknown viruses or unknown environmental marine viruses (45.64% of the assigned viral sequences) were removed from the analysis of viral communities’ diversity. Composition of the viral communities was represented at the family level using graphic tools of the *ggplot* package in RStudio (R version 3.5.3).

### 2.5. Statistical Analysis

The effect of tuna species, sex, sampling site and organs on the composition of viral communities was determined by a one-factor PERMANOVA with 999 permutations on Bray–Curtis matrix, using the “adonis” function of the *vegan* package [15]. For all tests statistical significance was assumed when *p* value < 0.05.

## 3. Results and Discussion

### 3.1. Tuna Host Specific Viral Niches

**Figure 1:**
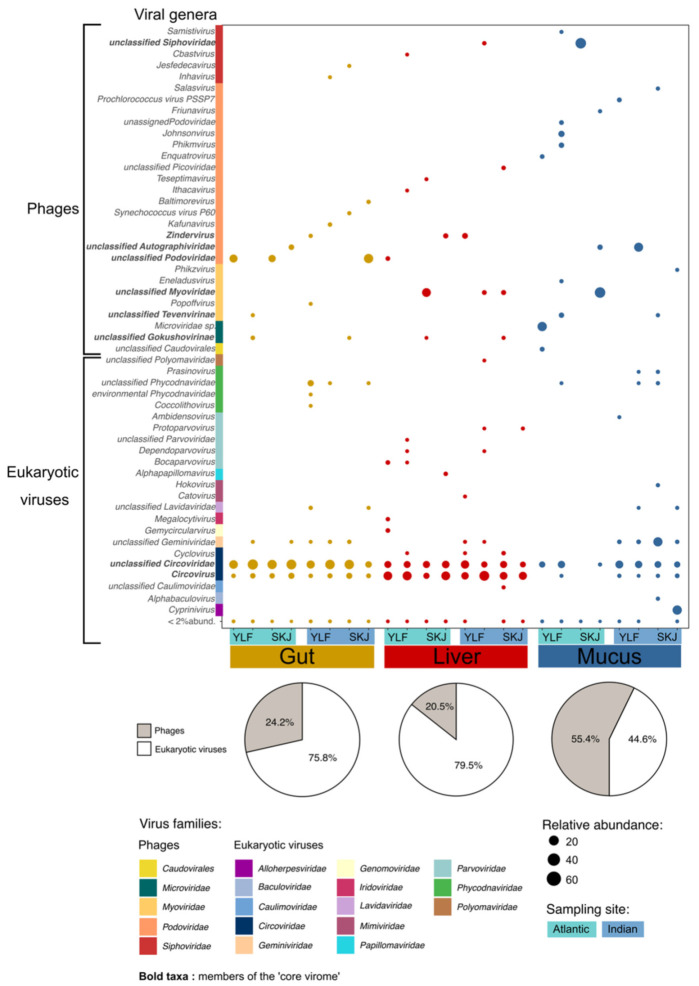
Relative abundance of viral genera in the gut, liver and skin mucus of yellowfin and skipjack tuna sampled in the Atlantic and Indian Oceans. Unknown and unclassified environmental viruses were removed from the analysis, thus, the calculation of relative abundance was based on the counts of each assigned contigs. Bold taxa were found within at least two organs and are thus members of the ‘core virome’. Pie chart represents the percentage of bacteriophage and eukaryotic virus contigs within each organ. These percentages were calculated based on the standardized relative abundance of assigned contigs within each organ.

The most striking finding was the discovery of a specific viral signature in the skin mucus, liver and gut, whatever the tuna species, sex or sampling site considered (Figure 1, Table 1). Interestingly, the proportion of bacteriophages varied greatly between the three organs, representing 55.4% of the assigned contigs in the skin mucus, but only 24.2% in the gut and 20.5% in the liver (Figure 1). Filipa-Silva et al. (2020) [8] also reported a substantial proportion of bacteriophages in the mucus layer of mackerel and seabream, but the digestive organs were not examined in their study. Our results are in line with the bacteriophages adherence to mucus (BAM) model, which postulates that animal mucosal surfaces harbor a high abundance of phages, which may act as a lytic barrier to limit the colonization of surrounding pathogens [1]. In this case, the prevalence of mucosal phages may indicate that tuna’s skin is certainly an essential component of its immune system.

Although the liver and gut are well-known bacterial niches in vertebrates [16], bacteriophages represented no more than 20% of the assigned contigs in our samples, suggesting that eukaryotic viruses may prevail in fish digestive organs [17]. This strongly differs from observations in other vertebrates such as birds and mammals, where phages were found to be predominant within the gut microflora [18,19,20]. However, Ramírez-Martínez et al. (2018) [21] also demonstrated that eukaryotic viruses were dominant in the fecal virome of various wild migratory duck species. Further studies are necessary to examine whether this is a particular feature in fish.

By analyzing the virome composition in the skin, gut and liver samples, we identified several organ-specific viral taxa (Figure 1). *Alloherpesviridae*, a well-known fish pathogen family responsible for skin lesions [22] and able to cause significant losses in salmonid, sturgeon and catfish aquaculture [23], was only detected in the skin virome. Most of the gut-specific genera identified belonged to the *Podoviridae* and *Siphoviridae* phage families, and to the *Phycodnaviridae* family for eukaryotic viruses. The former are common in marine environments [24] and have been previously observed within the gut virome of various terrestrial [16,25] and marine organisms [5,7,8]. The latter are known to infect algae [26] and have been previously identified in coral and fish viromes [5,8]. Since tuna are carnivorous, the detection of algal viruses in their gut is likely due to the presence of microalgae, either directly swallowed during food and water intake, or indirectly through prey containing algae themselves. Interestingly, the liver exhibited the greatest diversity of unique eukaryotic viruses, including *Cyclovirus*, *Megalocytivirus* and several genera of *Parvoviridae*, recognized as severe fish pathogens [27,28]. *Papillomaviridae*, well-known as human and mammal pathogens, were detected in the tuna liver—they have also been found in gilthead seabream, where they co-occurred with *Iridoviridae* and *Polyomaviridae* [29]. One should note that despite similar treatments, the type of samples might have induced a bias in the analysis of viral communities, for organic tissues of the different samples might have reacted differently to the sample preparation proceedings.

### 3.2. The Tuna ‘Core Virome’

While the virome composition was highly variable between the three organs, the findings also revealed the presence of several common taxa shared across the three organs. This ‘core virome’ was mainly composed of viral genera belonging to the *Myo*-, *Podo*-, *Sipho*- and *Microviridae* families, as well as genera of *Circoviridae* and *Geminiviridae* (Figure 1). The presence of a core virome composed of *Caudovirales*, *Microviridae*, *Hepadnaviridae* and several RNA viruses has been previously reported in the gills and liver of horse mackerel and gilthead seabream [8]. Circulation of bacteriophages has been previously observed in mammals [1], thus the presence of a core virome within tuna might suggest a form of active circulation of microorganisms within the virome, which should be further investigated to understand its mechanisms as well as its role in the health of the host.

### 3.3. Tuna Virome Composition Is Not Governed by Species or Sex

Our results demonstrated that whatever the organ considered, the composition of the virome did not differ between the two species, nor sexes (Table 1). Adult skipjack and juvenile yellowfin tuna (the size classes sampled in our study), whether male or female, are very similar anatomically, physiologically and behaviorally [30]. They also share the same habitat in the water column [31], and usually feed on the same prey (i.e., mostly fish, crustaceans and cephalopods) [32]. These similarities might explain the homogeneity of the virome between the two species. It may also be assumed that the bacteriophages observed within the intestinal and hepatic virome are generalist phages, able to infect different bacterial genera ensuring similar functions within the microbiome.

In line with the strong convergences between male and female tuna mentioned above, skipjack and yellowfin do not show sexual dimorphism and are distinguished only by the gonads [33]. We know that a few metabolic and hormonal differences exist between the sexes, notably during the reproductive period [32], however, our results suggest that these differences do not appear to be critical in influencing the composition of the virome in any part of the fish.

### 3.4. The Skin Virome Differs between Oceans

The skin mucosal virome of both skipjack and yellowfin varied significantly across the two sampling sites (Table 1). The variability of the surface microbiome with environmental conditions is well known in marine organisms [34], including the bacteriome of various fish species [35], but also the virome of corals [5] and sponges [7]. It is well established that the plasticity of the surface microbiome plays an essential role in the immune system of the host [34]. Thus, factors such as the composition of the surrounding planktonic communities, temperature or pH are likely to induce variation in the skin microbiome in order to effectively preserve the health of the host organism [34]. While studies focusing on distantly related fish have observed a species-specific composition of the surface microbiome, its homogeneity between skipjack and yellowfin tuna might also be explained by their physiological and behavioral similarities and their phylogenetic closeness.

## 4. Conclusions

These results allow a pioneering characterization of the tuna meta-virome and provided new knowledge on the microbiome of marine organisms. Demonstrating the remarkable diversity of viruses associated with tuna, these findings raise questions about the role and implication of these viruses in the health of the host. Public health questions are also emerging as to whether these viruses can affect the health of fish consumers.

## Figures and Tables

**Table 1:** Effects of the organ, sampling site, tuna species and sex on the diversity of viral communities assessed with permutational ANOVAs (PERMANOVA, 999 permutations) based on Bray–Curtis dissimilarity matrices. Bold values indicate a significant effect of the tested factor (*p* < 0.05).

	Organs	Ocean	Species	Sex
All samples	***p* = 0.001**	*p* = 0.138	*p* = 0.718	*p* = 0.999
Skin		***p* = 0.023**	*p* = 0.884	*p* = 0.863
Gut		*p* = 0.122	*p* = 0.159	*p* = 0.919
Liver		*p* = 0.136	*p* = 0.308	*p* = 0.972

## Data Availability

Not applicable.
